# Biological Factors, Metals, and Biomaterials Regulating Osteogenesis through Autophagy

**DOI:** 10.3390/ijms21082789

**Published:** 2020-04-17

**Authors:** Viviana di Giacomo, Amelia Cataldi, Silvia Sancilio

**Affiliations:** 1 Department of Pharmacy, University “G. d’Annunzio”, 66100 Chieti-Pescara, Italy; amelia.cataldi@unich.it; 2 Department of Medicine and Ageing Sciences, University “G. d’Annunzio”, 66100 Chieti-Pescara, Italy; silvia.sancilio@unich.it

**Keywords:** autophagy, osteogenesis, bone regeneration, osteoclastogenesis, biomaterial, osteoclast, oxidative stress, aging, cell survival, osteoblast

## Abstract

Bone loss raises great concern in numerous situations, such as ageing and many diseases and in both orthopedic and dentistry fields of application, with an extensive impact on health care. Therefore, it is crucial to understand the mechanisms and the determinants that can regulate osteogenesis and ensure bone balance. Autophagy is a well conserved lysosomal degradation pathway, which is known to be highly active during differentiation and development. This review provides a revision of the literature on all the exogen factors that can modulate osteogenesis through autophagy regulation. Metal ion exposition, mechanical stimuli, and biological factors, including hormones, nutrients, and metabolic conditions, were taken into consideration for their ability to tune osteogenic differentiation through autophagy. In addition, an exhaustive overview of biomaterials, both for orthopedic and dentistry applications, enhancing osteogenesis by modulation of the autophagic process is provided as well. Already investigated conditions regulating bone regeneration via autophagy need to be better understood for finely tailoring innovative therapeutic treatments and designing novel biomaterials.

## 1. Introduction

Autophagy is a complex dynamic process of recycling of non-essential or damaged organelles and proteins for nutrients and/or energy generation. Initially believed to be a mere way of transporting intracellular components to lysosomes, it is now known for playing an important role in maintaining cell homeostasis and survival under stressful conditions. Three types of autophagy with distinct regulatory mechanisms have been described: chaperone-mediated autophagy, microautophagy, and macroautophagy. Among these, macroautophagy is the most extensively studied because it is the most involved in cell biology, physiology, and disease.

Macroautophagy, henceforth referred to as “autophagy”, mainly involves the sequestration of cytoplasmic contents in a double-walled membrane followed by the fusion with the lysosomes. The lysosomal enzymes facilitate the degradation of the sequestered products. Autophagy is regulated by a group of evolutionarily conserved genes named Atg (autophagy related genes). The *Atg* genes have diverse functions, including the coordination of intracellular communication with all kinds of signaling pathways, even non-autophagic ones [1]. Autophagy has been shown to be essential for the maintenance of long-lived cells, such as neurons, cardiomyocytes, and osteocytes [2]. 

Osteocytes are the most abundant cell type in bone. They originate from osteoblasts that have undergone terminal differentiation during bone formation and subsequently have been engulfed by the extracellular matrix. Osteoblasts develop from pluripotent mesenchymal stem cells and are responsible for the formation of new bone, a process called osteogenesis. They produce bone by synthesis and secretion of collagen type I and aid the mineralization of the bone matrix. Hydroxyapatite (HA) constitutes most of the inorganic component of bone tissue. A third type of bone cells are osteoclasts, large multinucleated cells capable of bone resorbing. Bone health and homeostasis are the result of a delicate balance between the activity of osteoblasts and osteoclasts [3].

The bone loss is a common side effect in many physiological and pathological conditions, including ageing, exposure to chemicals and various diseases, such as osteoporosis. In addition, the reconstruction of large bone defects represents an extraordinary challenge both in orthopedics and dentistry. Biomaterial design and manufacturing requires a balanced combination of biochemical, biophysical, and material science concepts to make them biocompatible [4]. Interestingly, the previous definition of biocompatibility, as the lack of toxic or injurious effects on biological systems, has been recently replaced by a more complex idea. The notion of biocompatibility is currently intertwined with that of bioactivity, meaning the ability of a biomaterial to generate the most appropriate beneficial cellular or tissue response in a specific situation [5]. One of the strategies used to achieve this goal is the functionalization of the biomaterial by linking to its surface molecules able to modulate the oxidative stress and inflammation that may occur [6,7], promoting, at the same time, cell proliferation, migration, and differentiation.

In the field of biomaterials, the disruption of the autophagic pathway is mainly seen as a preferential target for nanoparticle-induced cytotoxicity in various tumor models [8,9,10]. However, autophagy activation has been found to be a key player in the cellular response against nano-toxicity, in non-cancerous cells [11,12]. Biomaterials designed for bone regeneration are discussed here for their ability to tune bone osteogenesis by the regulation of the autophagic process.

Autophagy is, indeed, highly involved in the metabolism of bone tissue. Multiple components of the autophagic pathway contribute to mediating the survival and functioning of the cells of the bone tissue, namely osteoblasts, osteocytes, and osteoclasts [13,14]. Increasing evidence suggests that an appropriate level of autophagy is associated with the survival of bone cells in many adverse conditions. Moreover, the autophagic process contributes to preosteoblast differentiation, osteoblast–osteocyte transition, and the genesis and functioning of osteoclasts [15].

It is not surprising, therefore, that research has long been focused on the role of autophagy in the homeostasis of the bone tissue, and, indeed, the mechanisms at the basis of both osteogenesis and autophagy have been extensively recently reviewed elsewhere [16,17]. 

Consequently, the authors decided to focus on different conditions that can affect osteogenesis by modulating autophagy in various experimental models. This review is therefore focused on the role of autophagy in the regulation of biological factors, metals, and biomaterial-related osteogenesis. Hence, the interest to investigate the effect of different stimuli on both processes, aiming to find a potential therapeutic alternative for many pathological conditions related to bone homeostasis. First, factors promoting and inhibiting osteogenesis through autophagy modulation are described. Dietary factors, mechanical stimuli, and metal exposition are described among the conditions upregulating osteogenesis. A focus on the stimuli inhibiting osteogenesis and related inflammation and oxidative stress is provided as well. A brief overview of the factors influencing osteoclastogenesis occurrence by autophagy modulation follows. Last, the authors focus on the importance of autophagy in bone regeneration driven by various biomaterials for application in both orthopedics and dentistry. 

## 2. Osteogenesis Enhancement

**Figure 1:**
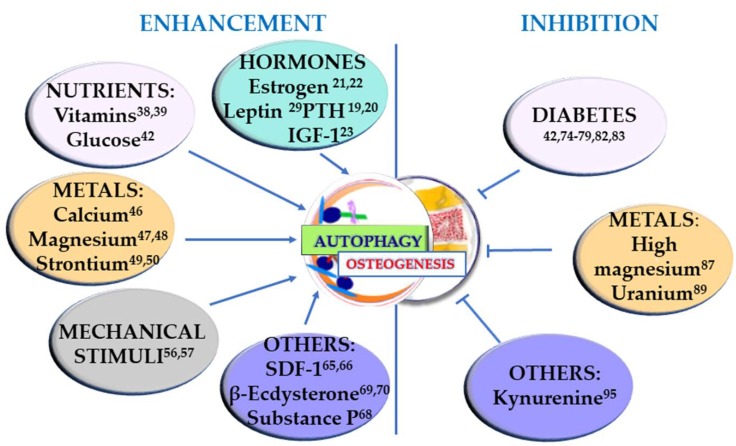
Stimuli that enhance osteogenesis through stimulation of autophagy (left) and conditions that negatively regulate osteogenesis by inhibiting autophagy (right). The superscript numbers refer to the references.

Osteogenesis occurs during the entire life of the bone tissue, participating in both modeling, i.e., the formation and shaping of bone, and remodeling, i.e., the replacement or renewal of old bone. It is also involved in bone healing following a fracture. Undifferentiated cells are also present in the bone, and they can be recruited to form osteoprogenitor cells and develop into osteoblasts. Osteoblasts produce an organic matrix, called osteoid, whose deposition is followed by its mineralization. Osteogenesis is therefore a complex, multi-step process which is finely regulated by many molecules and conditions. In this section, the factors that can positively regulate osteogenesis, through the involvement of autophagy, are taken into consideration and summarized in Figure 1 (left).

### 2.1. Hormones

Many systemic and local hormones can influence bone growth and remodeling. Indeed, bone homeostasis is related to the correct functioning of a number of systemic or circulating hormones that respond to changes in blood calcium and phosphorus concentrations [18]. Three calcium-regulating hormones play an important role in producing healthy bone: (1) parathyroid hormone or PTH, which stimulates both resorption and formation of bone and maintains the blood level of calcium, (2) calcitriol, derived from vitamin D, stimulates the intestines to absorb enough calcium and phosphorus and also affects bone directly, and (3) calcitonin, which inhibits osteoclast activity and reduce the levels of calcium in the blood. Previous studies have shown that PTH can promote autophagy in osteoblasts and chondrocytes and can also alleviate osteoarthritis by activating autophagy in articular chondrocytes [19]. In addition, in the MLO-Y4 osteocyte cell line, PTH upregulated the expression of the two autophagic markers LC3-II and Beclin-1, and decreased the level of Caspase-3, a hallmark of the apoptotic process [20].

There are no studies on autophagy mediating the effects of calcitonin or calcitriol. The latter, however, is in same way treated in Section 2.2, where its precursor, vitamin D, is discussed.

Sex hormones are also extremely important in regulating the growth of the skeleton and maintaining the mass and strength of bone, both in men and women. The female hormone estrogen have long been known to be positive regulators of bone homeostasis, enhancing osteocyte viability and promoting bone formation. Their sudden decrease is the main cause of osteoporosis in post-menopausal women. A recent study clarifies the mechanism of action of estrogen in differentiating human osteoblasts and their precursors, the mesenchymal stem cells (MSCs). Estrogen reduced apoptosis by promoting autophagy, thus contributing to osteoblast longer lifespan and mineralization capacity, via upregulation of RAB3GAP1, a complex that regulates the GTPases [21]. Florencio et al. suggested that estrogen maintains osteocytes viability, whereas its deficiency induces osteocytes apoptosis. The anti-apoptotic effect of estrogen on osteocytes may be related to autophagy regulation [22].

Growth hormone from the pituitary gland is also an important regulator of skeletal growth. It acts by stimulating the production of another hormone called insulin-like growth factor-1 (IGF-1), which can be produced also by bone tissue. IGF-1 binding to its binding protein 2 (IGFBP-2) stimulated osteoblast differentiation through induction of AMP-activated protein kinase (AMPK), a key sensor of cellular energy status. AMP-regulated osteoblast differentiation was finely tuned over time and is linked to the autophagic process. Early induction of AMPK in response to IGF-I/IGFBP-2 followed by suppression was required for osteoblast differentiation. Inhibition of AMPK influenced three autophagic markers: the ULK-1 phosphorylation as well as beclin-1 and microtubule-associated protein 1A/1B light-chain phosphatidylethanolamine conjugate (LC3II) induction. Direct inhibition of autophagy inhibited differentiation [23]. The ULK1 serine threonine kinase complex (involving also FIP200) plays a major role in autophagy initiation, whereas Beclin 1 and class III phosphatidylinositol 3-kinase (PI3KC3) complexes generate phosphatidylinositol 3-phosphate (PI3P) to act in autophagosome nucleation.

Cortisol, one of the hormones produced by the adrenal gland, has complex effects on the skeleton [24]. Small amounts are necessary for normal bone development, but large amounts block bone growth. Synthetic forms of cortisol, called glucocorticoids, are used as therapeutic treatment in many diseases. One of their main side-effects is osteoporosis, resulting from their ability to activate osteoclasts. This is discussed in Section 4.2.

Thyroid hormones increase the energy production of all body cells, including bone cells. They increase the rates of both bone formation and resorption but there is no evidence that their effects on bone tissue are achieved through the autophagic pathway. 

Another circulating hormone important for bone growth is insulin. The response to other factors that stimulate bone growth is impaired in individuals with insulin deficiency [25,26]. The latter being a condition that is a keystone in diabetic patients, and will be discussed in Section 3.1.

Finally, leptin, a hormone produced by fat cells, has also been shown to have positive effects on bone [27,28], and indeed it was found able to protect mesenchymal stem cells from apoptosis by inducing autophagy. In addition to AMPK, the serine/threonine kinase mTOR (mechanistic target of rapamycin), a master regulator of the canonical autophagic response of cells to nutrient starvation, appears to be involved [29]. 

### 2.2. Dietary Nutrients

The positive effects of dietary nutrients are largely correlated with autophagy in cancer, neurodegeneration, and many other pathological conditions [30,31]. Vitamins in particular are regulatory of autophagy in various situations, ranging from ocular disease to cancer and disorders of the digestive systems [32,33,34,35,36].

Among the others, vitamin D is involved not only in immune responses, anti-inflammation, anti-infection, and cancer prevention, but mainly in mineral and bone homeostasis [37]. Its active form, 1a,25-(OH)2D3 (vitamin D3) proved to have a dual effect on osteoclastogenesis by regulating autophagy, suggesting that some drugs targeting autophagy may act as an effective supplement of 1a,25-(OH)2D3 in treating osteoporosis [38]. Vitamin K2 also exerted a protective effect during osteoporosis by promoting osteoblast differentiation and mineralization and it has been recently demonstrated to stimulate autophagy in doing so, confirming this process as a potential therapeutic target [39].

A positive effect on osteogenesis can be achieved also by negatively regulating osteoclastogenesis. Indeed puerarin, a phytoestrogen extracted from *Pueraria lobata*, exerted its significant bone-protective effect by inhibiting the osteoclast precursor autophagy. Depending on the absence or presence of RANKL, puerarin reduced osteoclast precursor proliferation or differentiation, respectively. Therefore, an autophagic mechanism underlies the well-known therapeutic properties of puerarin in treating osteoporosis [40].

Glucose is a nutrient whose metabolism is closely associated to bone tissue homeostasis. Osteocalcin (OCN), a proteic hormone specifically expressed in osteoblasts and released into the circulation, may regulate glucose homeostasis, but, more importantly, high concentration of glucose can cause bone fragility [41]. Indeed, osteoporosis is a major complication for diabetes mellitus (DM) and the interlink among bone impairment, high glucose concentration, and autophagy is discussed in Section 3.1. Advanced glycation end products (AGEs) are proteins or lipids that become glycated as a result of exposure to sugars. They are a biomarker implicated in aging and the development of many degenerative diseases, including diabetes. AGEs and their receptor RAGE are usually associated with the development and progression of diabetes-associated osteoporosis, as well. Anyway, Meng and collaborators [42] found that AGE-modified bovine serum albumin (AGE-BSA) induced a biphasic effect on the viability and function of hFOB1.19 osteoblastic cells. Low doses (150 mg/L) and short exposure (up to 48 h) of AGE-BSA, increased cell proliferation and osteogenic markers expression, namely the soluble glycoprotein osteoprotegerin (OPG), the enzyme alkaline phosphatase (ALP), and OCN. The stimulation of both cell viability and osteogenic function were regulated by the Raf/MEK/ERK signal pathway and related to autophagy.

### 2.3. Metal Ions 

Metals represent another category of substances that can profoundly affect osteogenesis [43]. They are indeed largely employed in regenerative medicine, and their use in biomaterials is reviewed in Section 5 (Biomaterials, autophagy and osteogenesis). Here we will discuss the positive effects of metal ions and autophagy on bone homeostasis. 

Calcium is the most abundant metal of the human body where it provides skeletal strength and serves as a reservoir for maintaining blood calcium levels in a physiological range [44]. As electrolytes, calcium ions play a vital role in the physiological and biochemical processes of organisms and cells. As a second messenger, calcium is able to activate or inactivate various regulatory proteins such as enzymes, transcriptional factors, or molecular chaperones. Calcium ions outside cells are important for maintaining the potential difference across excitable cell membranes, protein synthesis, and bone formation. 

Calcium has been implicated in autophagic signaling pathways encompassing both mTOR and AMPK. Numerous studies have shown that cytosolic calcium signals can trigger autophagy. Moreover, there is evidence that buffering calcium affects not only the triggering of autophagy, but also proximal and distal steps during autophagic flux. However, calcium plays an essential role not only as a pro-autophagic signal, but can exert anti-autophagic actions too. For example, the sequestration of calcium by mitochondria during physiological signaling appeared necessary to maintain cellular bio-energetics, thereby suppressing the AMPK-dependent autophagy [45]. 

Calcium and inorganic phosphorus (present in biological systems as phosphate) are the ionic components required for hydroxyapatite formation during the mineralization of the extracellular matrix in bone tissue. The autophagic process has been demonstrated to be induced in osteoblasts during mineralization both in vitro and in vivo. The knockdown of autophagy-essential genes and osteoblast-specific autophagy-deficient mice demonstrated that autophagy deficiency reduces mineralization capacity. Moreover, it was suggested that autophagic vacuoles could be used as vehicles in osteoblasts to secrete apatite crystals [46].

Magnesium is the fourth most abundant metal ion in the body mostly stored in the skeleton and a natural agonist of calcium. It therefore plays a crucial role in bone metabolism and in the regulation of bone cells. The upregulation of two magnesium transporters during osteogenic differentiation has been recently demonstrated. Silencing either one accelerated osteogenic differentiation, partly through the activation of autophagy, underpinning the contribution of magnesium to autophagy and osteoblastogenesis [47,48]. It is worth noting that these two studies investigated the modulation of magnesium transporter during physiological osteogenesis. Magnesium exposure above the physiological value results in inhibition of the osteogenic process and it is discussed in Section 3.2. Strontium is an alkaline earth metal, which is already known for improving bone formation and suppressing bone resorption, resulting in increased bone apposition rates and bone mineral density [49]. In a recent article, the mechanisms underlying such effects were clarified. Cheng and collaborators [50] demonstrated that osteogenic differentiation induced by Sr was attenuated when cell autophagy was inhibited. This finding suggests that autophagic events in the osteobastic cell line MC3T3-E1 are essential in terms of strontium-induced osteogenic differentiation process. Elemental metal nanoparticles like cadmium and silver are known to cause oxidative stress and to be highly toxic [51] and indeed they will be addressed in Section 3 (Osteogenesis inhibition). Yet the exposure of human periodontal ligament progenitor cells to gold nanoparticles (AuNPs) induced upregulation of antioxidants, stress response genes, and autophagy as a cellular defense mechanism against oxidative stress toxicity [52].

### 2.4. Mechanical Stimuli

The study of the influence of mechanical stimuli on the structure of bone has long been a topic of scientific interest. Osteocytes have been defined as mechanosensory cells within the bone [53]. Osteocytes coordinate the remodeling process by the conversion of external mechanical forces into biochemical responses: a process called mechanotransduction [54]. During mechanotransduction, osteocytes acts like sensory cells within the bone, and their response is mediated by strain-derived fluid flow shear stress through the lacuno-canalicular network. Osteocytes will respond to this mechanical stimulus by opening ion channels and increasing the levels of intracellular calcium and protein kinase C, which consequently stimulate the release of potent anabolic regulators of bone growth, such as nitric oxide and prostaglandin E2 [55]. Interestingly, mechanical stimuli in bone tissue can regulate autophagy. Mechanical stretching, known to be able to promote the differentiation of bone marrow mesenchymal stem cells (BMSCs) to osteoblasts, was found to be related to autophagy. Its activation ameliorated hindlimb unloading-induced bone loss, by promoting osteoblast differentiation and consistent bone formation in a murine model [56]. The role of physical exercise in inducing osteogenic differentiation was confirmed by another study that found the modulation of osteogenic gene expression during physical activity. The expression of most osteogenesis-related genes, namely, *Runx2*, *Msx1*, and *Spp1*, appeared upregulated after running. RUNX2 (runt-related transcription factor 2), the master regulator of osteogenesis, acts early to commit mesenchymal stem cells to the osteochondral lineages and then induces the expression of collagen type I alpha 1 chain (COL1A1), which is crucial for the osteogenic phenotype. The genes belonging to the *Msx* (Msh homeobox) family are abundantly expressed at sites of inductive cell–cell interactions in the embryo, suggesting that they have a pivotal role during early development. *Ssp1* is the gene encoding for the protein osteopontin (OPN), also known as bone sialoprotein (BSP), a protein synthesized by bone cells to modulate matrix mineralization. Moreover, a positive correlation between *Atg3* and *Ulk1* gene expression and *Sox9*, encoding a protein involved in chondrocite differentiation, and *Runx2* gene expression in circulating progenitors were observed following physical exercise. Therefore, it could be assumed that the increased expression of chondrogenic and osteogenic genes is due to enhanced autophagy [57].

### 2.5. Direct and Indirect Proof

A direct link between autophagy and osteogenesis is represented by the use of the autophagy activator rapamycin in two different models. In the first, aging BMSCs exhibited degenerative changes, including imbalanced differentiation and reduced proliferation during aging, that contributed to age-related bone loss. Rapamycin could restore the biological properties of aged BMSCs by increasing osteogenic differentiation and proliferation capacity and decreasing adipogenic differentiation [58]. However, the supplementation of the diet with rapamycin offered no benefit in a model of osteogenesis imperfecta [59]. On the other hand, another strong correlation between autophagy and osteogenesis came from the demonstration that BMP-2-induced osteoblastic differentiation depends on the induction of the autophagic related gene *Atg7*, an essential regulator of autophagosome assembly [60]. In addition, another paper [61] reported that mice lacking the same autophagy related gene *Atg7*, had impairment in skeletal homeostasis. They had low bone mass and fractures associated with reduced numbers of osteoclasts and osteoblasts. *Atg7* silencing suppressed autophagy, reduced the amount of osteocyte cellular projections, and led to retention of endoplasmic reticulum (ER) and mitochondria in osteocytes.

The relevance of autophagy in bone regeneration was also found in an in vivo model of rabbits treated with implantation of tissue-engineered bone and injection of different concentrations of angiopoietin 2 in the defected bone site [62]. The growth factor promoted neovascularization in tissue-engineered bone and the repair of bone defects in a dose-dependent manner, which involved induction of autophagy. In this case the impact on osteogenesis was indirect, the effect being exerted on angiogenesis, that, in turn, favors bone regeneration. However, these findings highlighted the importance of autophagy in the complex multi-step process of bone formation.

Another indirect proof of the significance of autophagy in osteogenesis could be found in an in vitro model of fibroblasts from osteogenesis imperfecta recessive patients exposed to 4-phenylbutyrate (4-PBA). 4-PBA, a well-known chemical chaperone, approved by the Food and drug Administation, as an ammonia scavenger for urea cycle disorders, alleviated cellular stress by restoring ER size, normalizing the expression of apoptotic markers and stimulating autophagy [63].

### 2.6. Others

The stromal cell-derived factor-1 (SDF-1), also known as C-X-C motif chemokine 12 (CXCL12), is a cytokine protein ubiquitously expressed in many tissues and cell types and that is important in stem and progenitor cell recruitment in tissue repair after injury. It was found able to increase and accelerate bone formation both in vitro and in vivo [64]. Interestingly a direct interaction of the SDF-1/CXCR4 signaling axis, and specifically the SDF-1β isoform, with autophagy in proliferation and survival of BMSCs was demonstrated [65]. Moreover, SDF-1α-loaded silk fibroin scaffolds induced matrix-formation and new dentin deposition accompanied by autophagy in dental pulp stem cells (DPSCs) [66]. 

Substance P (SP), released predominantly by the peripheral terminal, is a conserved undecapeptide and a member of the tachykinin peptide family that acts as a sensory neurotransmitter and neuromodulator. Similar to growth factors, increasing studies have demonstrated that neuropeptides are critical for maintaining tissue homeostasis and SP has been demonstrated to have an osteogenic effect on BMSCs [67]. A recent study indicated that SP could promote osteogenic differentiation by activating autophagy in the same cell type [68]. In parallel, autophagic activity played an important role in restricting the excessive reactive oxygen species (ROS) generation and in mediating SP-enhanced BMSC osteogenic differentiation.

β-Ecdysterone is a naturally occurring estrogen analog derived from *Achyranthes bidentata* and *Cyanotis arachnoidea*. Multiple uses have been reported for this molecule, including similar protective effects to estrogen, which is the primary therapeutic strategy for the treatment of osteoporosis. 

BMSCs induced to osteoblastic differentiation were treated with dexametazone to study glucocorticoid-induced osteoporosis. The osteogenic markers ALP, RUNX2, and OCN were decreased, along with the expression levels of the autophagic regulators Beclin-1, autophagy protein 5, and microtubule-associated protein 1 light chain 3 II. The effects on cell differentiation and autophagy induced by dexamethasone were reversed by β-ecdysterone in a dose-dependent manner [69]. Similar results were obtained in vivo: in a murine model of osteoporois, β-ecdysterone was able to inhibit apoptosis through the induction of the autophagic process [70].

## 3. Osteogenesis Inhibition

**Figure 2:**
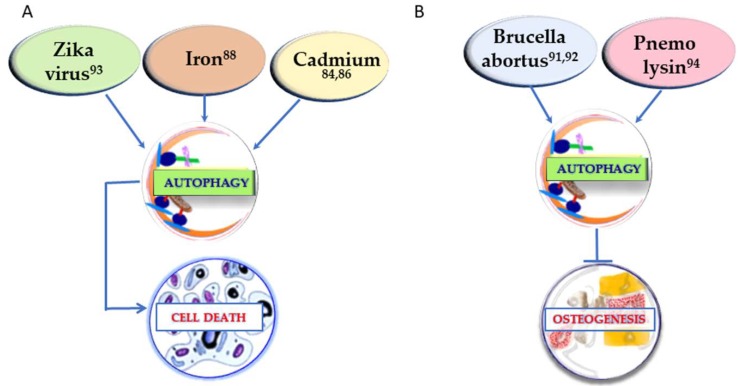
Conditions affecting osteogenesis by inducing cell death through upregulation of autophagy (**A**) and factors impairing osteogenesis by stimulation of the autophagic process (**B**). The superscript numbers refer to the references.

In order to have an extensive understanding of the factors that can regulate osteogenesis through autophagy, it is crucial to take into consideration also the conditions that show a negative regulation of osteogenesis through autophagy. If the positive regulation of osteogenesis is generally linked to autophagy stimulation, in osteogenesis inhibition the mechanism may vary. In Figure 1 (right) the substances that inhibit both osteogenesis and the autophagic process are reported. In Figure 2 the other two different strategies of action of the determinants presented in this section are described. In Figure 2A, the conditions upregulating autophagy and leading to cell death are summarized, whereas in Figure 2B the agents leading to osteogenesis inhibition by autophagy enhancement are represented.

### 3.1. Diabetes

In addition to other well-known complications, type 2 diabetic patients also have fragile bones caused by faulty mineralization, mainly due to increased adiposity among diabetic patients that affects both osteoblast and osteoclast functions. Other factors that increase fracture risk in diabetic patients are augmented oxidative stress, inflammation, and drugs administered to treat diabetes [71]. Long-standing diabetes causes disruption of the bone marrow microenvironment by depleting and altering stem/progenitor cells resulting in enhanced adipogenesis and depressed osteogenesis [72,73]. On the basis of the results from a streptozotocin-induced diabetic rat model, BMSCs were grown in a hyperglycemic medium. They underwent an autophagy mechanism, and diverted from an osteogenic to a metabolically stressed adipogenic phenotype with production of a monocyte-adhesive hyaluronan matrix. The latter could be the mechanism involved in the osteopenic response of streptozotocin-treated diabetic rats [74]. Another study found that BMSCs from type 2 diabetes mellitus patients (DM-BMSCs) showed decreased osteogenic differentiation and autophagy level, and increased senescent phenotype. The same type of cells from healthy donors exposed to hyperglycemic and hyperinsulinemic conditions showed phenotypes similar to those of DM-BMSCs. In summary, insulin impeded osteogenesis of BMSCs by inhibiting autophagy and promoting premature senescence, with the involvement of the TGF-β1 pathway, notoriously related to cell differentiation [75]. Consistent with these findings, the early induction of AMPK in response to IGF-1/IGFBP-2, by activating autophagy, is required for osteoblast differentiation as already suggested by another research group [76]. Insulin-like growth factor 1 is a potent stimulant of osteoblast proliferation and recent studies showed that a member of the insulin-like growth factor binding protein family, IGFBP-2, was also required for optimal IGF-1-stimulated osteoblast proliferation and differentiation [77]. These findings suggested that these early catabolic changes were important for determining the energy source for osteoblast respiration. Downregulation of these components could be required for induction of glycolysis, which is required during the final anabolic stages of differentiation [23]. 

Advanced glycation end products (AGEs) are a group of heterogeneous compounds that accumulate in the bone tissue of diabetic patients. In a study, AGEs increased apoptosis in the osteoblastic cell line MC3T3-E1. At the same time, the autophagy was upregulated as represented by an increase in the total LC3 level and the LC3II/LC3I ratio, and a decrease in the expression of p62/SQSTM1, a biomarker of the degradation of autolysosomes with its expression negatively correlated with autophagy level. The further induction of autophagy by administration of rapamycin attenuated AGE-induced apoptosis. Interestingly, blunting the oxidative stress with the antioxidant N-acteylcysteine, suppressed autophagy. Autophagy hence played a protective role in MC3T3-E1 cells during AGEs-induced apoptosis, and ROS were essential in upregulating AGEs-induced autophagy [78]. AGEs were already reported to trigger osteogenesis through autophagy at low concentrations (see Section 2.2). However, in the same paper [42], it was demonstrated that increasing AGE concentration (200 mg/mL) and exposure time (72 h) resulted in decreased cell proliferation and osteogenic functions in hFOB1.19 cells.

Since many studies have shown that a high glucose environment can impede periodontal ligament stem cells (PDLSC) proliferation and differentiation ability and affect the regeneration of periodontal tissue [79,80], in another model of diabetic rats the role of autophagy in this process was investigated. Fluctuations in many autophagy and osteogenesis related markers implied that autophagy was involved in the osteogenic process and that high glucose weakened physiological functions in PDLSCs, including osteogenesis and autophagy. Remarkably, regulation of autophagy could partly recover the cells’ osteogenic abilities both in vitro and in vivo [81]. 

To complete the picture, it is crucial to mention a study about the effect of melatonin on type 2 diabetes osteoporosis. This hormone, also employed as pharmaceutical treatment, could suppress autophagy, enhance bone microstructure, and promote osteoblast osteogenesis, by downregulating the ERK pathway in type 2 diabetic osteoporosis and in hFOB 1.19 osteoblasts treated with high glucose [82].

### 3.2. Metal Ions

As already mentioned above, metals as cadmium can be toxic for cells involved in osteogenesis. To this regard, two papers from the same group are consistent in demonstrating autophagy induction following cadmium exposure in mouse bone marrow mesenchymal stem cells. The first [83] found that cadmium increased both mRNA and protein expression of FOXO3a, a member of the forkhead-box (Fox) family of transcription factors, which plays an evolutionarily conserved role in cell proliferation and survival in a variety of tissues. In addition, AMPK was demonstrated to enhance FOXO3a nuclear translocation and transcriptional activity. These results demonstrated that overactivated autophagy may be the primary contributing factor underlying cadmium-induced MSC death. However, since the *Foxo3* knockdown could not completely prevent cadmium-induced autophagy, in a more recent paper other pathways were investigated [84]. Transcription factor E3 is a member of the basic helix-loop-helix leucine zipper family of transcription factors, and has recently been identified as a master regulator of the expression of genes that are associated with autophagy and lysosomal biogenesis [85]. Transcription factor E3 was found to play a role in cadmium-induced autophagic cell death in MSCs, independently by MTORC1.

In the Section 2 (see Section 2.3) physiological levels of magnesium were already described to be crucial for healthy osteogenesis, this metal acting as a calcium antagonist and preventing aberrant ossification. It is however valuable to point out that, at high doses, magnesium can impair the process of osteogenesis. Matrix mineralization, expression of collagen type I, and the mineral crystals growth in human bone marrow-derived mesenchymal stem cells can be suppressed by high magnesium (1 mM). The upregulation of autophagy by ATP reverted the effects of high magnesium on extracellular mineralized matrix deposition [86]. 

The divalent metal transporter 1 (DMT1) is a 12-transmembrane-domain protein found in a range of tissues, including bone, on which the cellular transport of iron ions is heavily dependent. It was previously found closely associated to osteoporosis, but in a recent paper the increased expression of DMT1 was found to induce iron overload. The iron accumulation in turn, induced osteoblast autophagy and apoptosis, thus affecting the pathological processes of bone loss [87].

Natural uranium (U), which is present in our environment, exerts a chemical toxicity, particularly in bone where it accumulates. In UMR-106 osteoblastic cell line, U(VI), the form uranium is found in atmospheric conditions and in most environmental systems, affecting mineralization function even at subtoxic concentrations. At the same time, the autophagic flux was impaired as a result. In addition, a reduced degradation of autophagic vesicles could lead to non-elimination of damaged mitochondria, resulting in enhanced ROS production which is one of the mechanisms of U(VI) toxicity in osteoblasts [88].

### 3.3. Pathogens

A little investigated regulation of osteogenesis through autophagy came from biological agents. First, bacteria of the genus *Brucella* are Gram-negative microorganisms that causes brucellosis, a disease that commonly results in persistent, chronic involvement of osteoarticular system which usually leads to bone damage [89]. *Brucella abortus* induced the activation of the autophagy pathway in osteoblast cells and this activation was involved in the impairment of osteoblast function and bone formation [90]. More importantly, it was demonstrated that *Brucella* infection uses the autophagic pathway to inhibit matrix deposition early during infection, while at later times the process of differentiation of osteoblasts takes control of the pathway, confirming that autophagy was required for osteoblast terminal differentiation [91]. Second, infection from the Zika virus (ZIKV), a mosquito-borne flavivirus, during gestation is deemed to be coupled to birth defects through direct impairment of neurogenesis. It has become an international health concern and has been declared as a public health emergency by the World Health Organization. Most relevant to the aim of this review, ZIKV infection caused aberrant cranial osteogenesis by greatly enhancing autophagy, which led to neural crest cells (the progenitor cells of bone formation in the skull) apoptosis [92]. Third, pneumolysin (PLY) is the main virulence factor of *Streptococcus pneumoniae* and a common cause of septic arthritis and osteomyelitis. As other toxins, PLY induced ROS production during osteoblast differentiation, leading to early upregulation of autophagy. The ROS-mediated regulation of AMPK and mTOR, which downregulated the expression of the transcription factor Sp1, resulted in an inhibition of differentiation in human osteoblast- like cells [93].

### 3.4. Kynurenine

Kynurenine, a tryptophan metabolite, is a key upstream mechanism that appears to target a number of osteogenic pathways with age. Physiological levels of kynurenine disrupted autophagic flux and autophagolysosomal production, inducing a senescent phenotype in BMSCs via Aryl hydrocarbon receptor (AhR) signaling, inducing downregulation of osteogenesis [94].

## 4. Osteoclastogenesis

Aim of the present review is to extensively summarize the literature on the interplay between autophagy and osteogenic differentiation. Indeed, recent studies have highlighted the influence of autophagy in osteoclast differentiation and function. The receptor activator of NF-κB ligand (RANKL) is involved in osteoclast differentiation [95]. During this process an increase of autophagic protein levels such as ATG5, ATG7, ATG4β, and LC3 was evident. These are the main proteins for autophagosome formation responsible for generating the osteoclast-ruffled border and the lysosomal secretion [96]. Moreover, the increase of LC3/ILC-3I ratio is related to p62 degradation, essential in the generation of the filamentous actin ring, a key feature of osteoclatogenesis [97].

A brief overview of the main factors influencing osteoclastogenesis is provided. Nevertheless, it is not meant to be an exhaustive reviewing of the literature on the subject.

### 4.1. High Glucose

If it is clear that high glucose negatively affects osteoblastogenesis, the role of high glucose in the physiology and differentiation of osteoclasts is still controversial. In the only study relating autophagy to osteoclast differentiation, glucose proved to negatively affect osteoclast formation and function but did not affect the proliferation of RAW264.7 cells. Suppression of the AMPK/mTOR/ULK1 signaling axis by high glucose decreased autophagy in differentiating osteoclasts, demonstrating that autophagy participates in osteoclast differentiation and function and can be inhibited by high glucose concentration [98]. 

### 4.2. Glucocorticoids

Glucocorticoids remain an effective therapy for many inflammatory/autoimmune disorders. Nevertheless, moderate-to-high doses of glucocorticoids or their prolonged administration lead to osteoporosis, characterized by consistent changes in bone remodeling with decreased bone formation as well as increased bone resorption [99]. 

Autophagy protects osteocytes from glucocorticoid-induced apoptosis, but past some threshold the process of autophagy leads the cells to apoptosis. Excess glucocorticoids impaired osteoblastogenesis by inducing Wnt antagonists, including *Dkk1*, *Sost*, *and sFRP-1* [100]. Lian et al. reported that HSP60 (heat shock protein 60) was required to sustain autophagic markers ATG4, and ATG12 expression, LC3-II conversion, and autophagic puncta formation. It also alleviated the glucocorticoid-induced loss of osteogenic gene expression and mineralized matrix accumulation via RPTOR signaling [101].

Interestingly, ROS, which play a crucial role in osteoclastogenesis, and autophagy flux activity were found upregulated consistently with the dose-dependent effects of the glucocorticoids on osteoclast formation and function. These results implied that with glucocorticoid administration, ROS and autophagy, as a downstream factor of ROS, played vital roles in osteoclast formation and function [102]. The same conclusions were found in an in vivo model of osteoporosis [103]. Taken together, the knowledge of the mechanisms at the basis of glucocorticoid-induced osteoporosis, suggests the use of autophagy as a target in this disease [104].

Consistent with these data, lipopolysaccharide (LPS) induced autophagy, osteoclastogenesis, and reactive oxygen species in bone marrow derived macrophages that were pre-stimulated with RANKL. Removal of ROS decreased LPS-induced osteoclast formation and autophagy as well [105]. A very recent paper reviewed the role in bone homeostasis of both autophagy and apoptosis induced by glucocorticoids [106].

### 4.3. Oxidative Stress

Since oxidative stress has long been linked to osteoclastogenesis enhancement, another study suggested that the differentiation of osteoclast precursors induced by monocyte chemotactic protein-1, a CC chemokine commonly found at the site of tooth eruption, is mediated via oxidative stress. The oxidative stress, in turn, caused ER stress leading to autophagy, revealing a novel mechanism in OC differentiation [107]. As oxidative stress and apoptosis are strictly related, already published data demonstrated that TNF receptor associated factor-6 (TRAF6)/c-Jun N-terminal kinase1 (JNK-1) prevented osteoclast precursor apoptosis and mediated autophagy, enhancing RANKL-induced osteoclastogenesis via TRAF3 degradation [108].

Oxidative stress is strictly associated to inflammation, which, in bone, leads to activation of osteoclasts and to the subsequent bone destruction [109]. The pro-inflammatory cytokine IL-17, already related to aberrant ossification in rheumatoid arthritis and osteoarthritis patients [110], is also associated to an elevated number of osteoclasts in periodontitis [111]. Two studies suggested that IL-17 was responsible for osteoclast differentiation and bone resorption, both in vitro and in vivo, via activation of autophagy. These effects of IL-17 were found both in primary mouse bone marrow macrophages [112] and osteoclast precursors through the activation of the RANKL-JNK pathway [113].

### 4.4. Microgravity

Microgravity is an uncommon situation in which bone loss is experienced during space flights. Osteoclasts and their precursors were already found to be the target of mechanical forces that could be responsible for modulating gene expression associated with osteoclast differentiation/activity [114]. During exposure to microgravity, an induction of autophagy was registered and proved to play an important role in enhanced osteoclast differentiation [115].

## 5. Biomaterials, Autophagy, and Osteogenesis

A biomaterial is any material (e.g., polymer, ceramic, metal, or composite) that has been engineered to interact with biological systems for a medical purpose, either a therapeutic (treat, augment, repair, or replace a tissue function of the body) or a diagnostic one. Biomaterials are used every day in dental and orthopedic applications, surgery, and drug delivery. They can be derived either from nature or synthesized in the laboratory using a variety of chemical approaches and materials. Biomaterials can be broadly categorized in metals, polymers, ceramics, and composite materials. This classification is followed in this section to discuss biomaterials promoting bone regeneration by modulating the autophagic process. The research in the field of biomaterials applied to bone regeneration is actually focused on the modifications of their surfaces in order to improve their bioactivity. In this perspective two strategies can be used: the functionalization of the biomaterial/cell interface by linking to its surface osteoinductive/osteoconductive molecules; and the modification of surface topography to make them more suitable for cell growth and differentiation. In the following paragraphs many examples of these strategies are given, relating them to the biomaterial used. 

Table 1 provides an overview of the biomaterials discussed in this section along with the experimental model they were tested and the signaling pathway involved (where applicable).

### 5.1. Polymers

Silicon based materials have long been studied for their application in regenerative medicine either for their proangiogenic role [116] or their use in scaffolds that mimic the structure and composition of bone tissue [117]. In this field, the synthesis of silicate-containing hybrids by the sol–gel method is a new route to preparing bioactive implants with improved mechanical properties. These materials can be degraded by the physiological environment, which involves the eventual bone colonization and full tissue restoring. Actually, the research is focused on tailoring the hybrid implants for bone tissue regeneration rather than bone substitution. Silicate-containing hybrids must promote the osteogenic performance of the osteoblast-like cells [118]. 

Interestingly, orthosilic acid, a unique soluble form of silicon, enhanced the BMP-2/RUNX2 and COL-1 protein expression in preosteoblastic cells, promoting differentiation and mineralization of osteoblasts through the activation of the autophagic pathway [119]. Moreover, an engineered bioactive silica-based nanoparticle formulation (NPs) was found able to stimulate in vitro differentiation and mineralization of osteoblasts and increased bone mineral density in young mice in vivo [120,121]. In the search of the mechanisms underlying such results, Ha and collaborators [122] found that the stimulation of autophagy and associated signaling suggests a cellular mechanism for the stimulatory effects of silica nanoparticles on osteoblast differentiation and mineralization. They notably suggested that it is the size of the nanoparticles (50 nm) that stimulates autophagy rather than the materials they are made of. These considerations are remarkably in line with what was found about gold nanoparticles discussed in Section 2.4. In the study cited above [52] the 45 nm AuNPS were the most effective in promoting both autophagy and osteogenesis.

Chitosan is a polysaccharide copolymer of glucosamine and N-acetylglucosamine derived by partial deacetylation of chitin from crustacean shells. Recently, many studies have investigated the effects of chitosan film or membrane on the morphology, stemness, and multi-differentiation abilities of MSCs. It has been demonstrated that MSCs cultured on chitosan film formed spheres and the expression of stemness marker genes increased significantly when MSCs were cultured using chitosan film compared with 2D monolayer culture systems [123]. More importantly, culture on chitosan film resulted in an increased differentiation potential of MSCs into mesenchymal lineages, such as osteoblasts [124]. In the same experimental model, mTOR signaling was activated especially in senescent cells, whereas its suppression or knockdown selected more primitive MSCs that are enriched in gene expression of pluripotency, in vitro osteogenesis, and in vivo bone formation [125].

### 5.2. Metals

#### 5.2.1. Titanium and Nanostructure

Most of the recent research on biomaterials is actually focused on titanium, the most often used material, due to its biocompatibility and mechanical properties, both for orthopedic and dentistry applications, in substitution of ceramics, polymers, and other metals [126,127]. 

In a lately published paper, an osteocyte-conditioned medium proved to inhibit osteoclast differentiation from bone marrow monocytes (BMMs) to osteoclasts. However, TiAl6V4 alloy particles (TiPs) attenuated this inhibitory effect by markedly decreasing the expression of IFN-β, an osteoclastogenesis-associated factor. Additional evidence suggested that TiPs decreased the expression of IFN-β in osteocytes via stimulation of autophagy [128].

Among the others, one distinctive strategy used to improve the bio-functionality for titanium implants, was the use of exosomes derived by macrophage stimulated with BMP2, that were already known for their beneficial effects on osteogenic differentiation [129]. The incorporation of BMP2/macrophage derived exosomes dramatically increased the expression of osteoblastic differentiation markers in MSCs. Remarkably, the pro-osteogenic role of the titanium nanotubes incorporated with BMP2/macrophage-derived exosomes is mediated by autophagy [130].

In the biomaterial field of research, it is already known that biomaterials with varied surface topography have more biocompatible features and better interactions with the surrounding living tissues. Rough surfaces caused osteoblast differentiation via the autophagic-dependent PI3/Akt signaling pathway. One surface provoked the development of a third population of small, granular cells, responsible for cell cluster formation, which were important for the formation of bone noduli and mineralization. When autophagy was inhibited, both mature osteoblasts and small cells were absent, and the cell cluster formation was also prevented. Autophagy therefore has to play an essential role in the osteoblast differentiation on titanium-based surfaces with rough topography [131].

The nanosized surface is well known for its ability to interfere with intracellular procedures and a nanotube structure was found able to enhance mTOR-independent autophagy in osteoblasts compared to a flat surface. Further analysis revealed that autophagy was temporally promoted by nanotubes in the initial day contact, and cell membrane stretching appeared to be the central regulation factor. The process was also reversible by exchanging the substrate nanotopographies in different cell lines. In summary, the nanotopographic surface is able to induce temporal and reversible autophagy, which may be used as a versatile method to control cell differentiation [132].

Implant topography is associated with the functionality of osteogenic transcription factors directed by β-catenin in the nucleus. This protein can be degraded by YAP (Yes-associated protein) which is susceptible to autophagic flux. Nanotopography, in comparison with smooth surfaces, was associated with higher β-catenin nuclear translocation, osteogenic differentiation, and autophagy, and less cytoplasmic YAP in MC3T3-E1 cells. These results demonstrated an involvement of this pathway in the osteogenesis observed in response to titanium implants [133].

#### 5.2.2. Alumina

The osteoimmune environment plays indispensable roles in bone regeneration because the early immune environment that exists during the regenerative process promotes the recruitment and differentiation of osteoblastic lineage cells [134]. Nanoporous anodic alumina with different sized pores had modulatory effects on macrophage responses and consequently on the osteogenic differentiation of BMSCs. The role of macrophages in osteogenesis was already suggested to be indispensable [135]. The effect of the 50 nm nanoporous alumina structures on macrophage spreading and shape resulted in osteogenic differentiation of BMSCs, improving the osteogenic capacity of bone biomaterials with a mechanism related to autophagy activation [136].

#### 5.2.3. Silver

Silver is used in a variety of medical and general devices for its antimicrobial properties. It is, therefore, widely used in the form of nanoparticles in medicine, in order to retard and avoid bacterial infection [137,138]. Despite their antimicrobial action, silver nanoparticles (AgNPs) lack toxicity towards eukaryotic cells, because of the induction of the autophagic process [12]. Interestingly, linking the silver nanoparticles to thermosets made of materials commonly used in the dental practice resulted in further reduced cytotoxicity [139], confirming that the adsorption of molecules on biomaterial surfaces can improve their biocompatibility. 

Many results were recently achieved regarding effects of AgNPs on osteogenesis of stem cells [140,141,142]. Again, the linking of AgNPs, whose potential toxicity raises serious concerns, on titanium surfaces proved to be a successful strategy [143]. Moreover, AgNPs activated autophagy and osteogenesis. The administration of the autophagy inhibitor 3-methyladenine could reverse both processes, binding the occurrence of osteogenesis to the autophagic activity in human MSCs [144].

### 5.3. Ceramics

Hydroxyapatite (HA) is a natural occurring mineral present in the human skeleton. In biomaterial applications it can be used in combination with alginate to study the improved osteoblast differentiation of DPSCs [145,146].

HA-nanoparticles (HANPs) promoted osteoblast differentiation in a dose-dependent manner the osteoblast cell line MC3T3E1. In addition, the internalized HANPs were located in typical autophagic vacuoles and increased the ratio of LC3II/LC3I, indicating HANPs induced cell autophagy. Moreover, the induction of autophagy was via the mTOR signaling pathway also in a concentration dependent manner. Collectively, these results revealed that HANPs modulates osteoblast differentiation by mediating autophagy in a dose-dependent manner [147].

Polydopamine-templated hydroxyapatite (tHA) is a type of nano-biomaterial, designed as an alternative to the traditional hydroyapatite (HA,) that can promote osteogenesis in bone tissue engineering. The reinforcement of polycaprolactone (PCL) matrix with tHA enhanced cell adhesion, spreading, and proliferation of human mesenchymal stem cells. More importantly, tHA nanoparticles exposed on the surface of composite nanofibers could further promote osteogenesis of human MSCs in vitro [148]. However, as already seen in other experimental systems, the concentration is crucial. Indeed, high concentrations of tHA stimulated ROS production, resulting in cell injury and apoptosis in PDLSCs. Nevertheless, the triggering of the AMPK/mTOR signaling pathway when tHA is in combination with metformin, led to autophagy activation and consequent increased viability of human PDLSCs with a further improvement of the osteogenic effect [149].

Interestingly, also the incorporation of fluorapatite (FA) crystals within the three-dimensional PCL nanofiber scaffolds provided a favorable extracellular matrix microenvironment for the growth, differentiation, and mineralization of human DPSCs [150]. In a different cellular model, the inhibition of autophagy at earlier stages (days 1 to 3) could affect human adipose stem cell (hASCs) osteogenic capability and mineralization when grown on PCL+FA scaffolds. These results suggested that autophagy was indispensable during the early stage of osteogenic differentiation in this model [151].

## 6. Conclusions

The health of the bone tissue is strictly related to the differentiation of osteoblasts, the cell responsible for the deposition of organic osteoid and matrix mineralization, which leads to osteogenesis. Autophagy is thoroughly involved in the development of these cells, contributing therefore to bone homeostasis. Impairment of autophagic activity leads to disruption of the bone-remodeling balance, which leads to pathological state and failure of biomaterial implants. Autophagy modulation has been shown to have an intriguing potential as target for ageing, biomaterial design, and the therapy of various pathological conditions. This review offers a deep insight in the mechanisms and stimuli driving osteogenesis in combination with autophagy, providing a useful tool for the development of innovative therapeutic strategies. As far as the authors know, this is the first review summarizing the role of autophagy in osteogenesis promoted by different types of biomaterials. The knowledge of the conditions improving biomaterial bioactivity will help future research to design new biomaterial solutions.

## Figures and Tables

**Table 1:** Biomaterials, experimental models, and signaling pathways.

Biomaterial	Model	Pathway	Reference(s)
Silicon, Orthosilic acid	Murine preosteoblast MC3T3-E1	BMP2/RUNX2 Col1	[116,117,118,119]
Silica NPs	Murine preosteoblast MC3T3-E1	ERK1/2, LC3, p62	[120,121,122]
Chitosan	Primary hMSCs	mTOR/S6K/S6/4E-BP1	[123,124,125]
TiAl6V4 particles	Osteocytic cell line MLO-Y4	IFN-β	[126,127,128]
Titanium	hBMSCs		[129,130]
Titanium	Human osteoblasts	PI3K/Akt	[131]
Titanium	Murine preosteoblast MC3T3-E1		[132]
Titanium	Murine preosteoblast MC3T3-E1	β-catenin/YAP	[133]
Alumina	rBMSCs	Wnt BMP	[134,135,136]
Silver NPs			[137,138,139,140,141]
Silver NPs	Mouse		[142]
Silver NPs	hMSCs		[143,144]
Hydroxyapatite	DPSCs		[145]
Hydroxyapatite	DPSCs	IL-6	[146]
Hydroxyapatite	Murine preosteoblast MC3T3-E1	mTOr	[147]
Hydroxyapatite	PDLSCs	AMPK mTOR	[148,149]
Fluorapatite	hASCs		[150,151]

## Abbreviations

4-PBA	4-phenylbutirrate
AGEs	advanced glycation end products
ASCs	adipose stem cells
BMMs	bone marrow monocytes
BMP	bone morphogenetic protein
BMSCs	bone marrow mesenchymal stem cells
DM-BMSCs	diabetes mellitus bone marrow mesenchymal stem cells
DMT1	divalent metal transporter 1
DPSCs	dental pulp stem cells
ER	endoplasmic reticulum
FA	fluorapatite
HA	hydroxyapatite
IGF-1	insulin-like growth factor 1
IGFBP	insulin-like growth factor binding protein
LPS	lipopolysaccharide
MSCs	mesenchymal stem cells
NPs	nanoparticles
PDLSCs	periodontal ligament stem cells
PCL	polycaprolactone
PLY	pneumolysin
ROS	reactive oxygen species
SDF-1	stromal cell-derived factor-1
SP	Substance P
ZIKV	Zika virus
